# Mechanisms Underlying Gender Influence on the Clinical Course and Immunopathogenesis of Systemic Lupus Erythematosus: An Explorative Review

**DOI:** 10.7759/cureus.73646

**Published:** 2024-11-13

**Authors:** Vamshi Varaganti, Sabitha Vadakedath, Jayashankar CA, Venkataramana Kandi, Pooja V B, Mir Hyder Hussain, Anuradha V, Kalidindi Gayathri

**Affiliations:** 1 Medicine, Prathima Institute of Medical Sciences, Karimnagar, IND; 2 Biochemistry, Prathima Institute of Medical Sciences, Karimnagar, IND; 3 Internal Medicine, Vydehi Institute of Medical Sciences and Research Centre, Bangalore, IND; 4 Clinical Microbiology, Prathima Institute of Medical Sciences, Karimnagar, IND; 5 General Medicine, Vydehi Institute of Medical Sciences and Research Centre, Bangalore, IND

**Keywords:** autoimmune disorder, clinical consequences, disorders, gender-based, immunopathogenesis, systemic lupus erythematosus

## Abstract

Systemic lupus erythematosus (SLE) is an autoimmune disorder with a complex clinical course and diverse presentations. The immunopathogenesis of SLE has long intrigued physicians and researchers. Despite its extensive global prevalence, there is no specific treatment to prevent and treat SLE, and in the majority of SLE patients, the management involves controlling disease remissions and symptom reactivations or flares. SLE patients suffer from damage to different organs of the body, complicating disease management. They are predisposed to infectious diseases that could contribute to enhanced disease progression. Devising effective management strategies requires a comprehensive understanding of the effects of the disease and its influence on the immune system. SLE affects females more frequently than men. However, male SLE patients often suffer from more severe disease than females. Gender variations have also been noted in clinical manifestations in patients with SLE. In light of this, additional research is needed to understand these variations and promote the progress of gender-specific patient management and treatment strategies.

This review aimed to compare the influence of gender on the clinical consequences, immunopathogenesis, and associated consequences between male and female SLE patients. An extensive literature search was conducted to collect relevant data. PubMed, MEDLINE, Embase, and Google Scholar were searched from inception to the present for articles that compared clinical outcomes and associated disorders in terms of gender among SLE patients. We also explored the immunopathogenesis, mechanisms underlying gender-based clinical effects of SLE, and infectious disease-related consequences. Additionally, we provide key updates regarding the treatment and management of SLE.

## Introduction and background

Systemic lupus erythematosus (SLE) is a quintessential systemic autoimmune disease characterized by multiorgan inflammation [1]. SLE is a clinically heterogeneous disease, making it difficult to classify and diagnose [2]. While the exact cause of SLE remains unknown, it is thought to involve a combination of genetic predisposition and environmental factors; also, immunological and endocrine systems play a role in the etiopathogenesis of SLE [3,4]. It occurs when the immune system wrongly targets its tissues, producing pathogenic autoantibodies that target the nuclear antigens [5]. These immune reactions lead to the formation of immunological complexes, and inflammation of various organs in the body [6]. The loss of immunological tolerance to self-antigens results in the development of pathogenic autoantibodies, which induce tissue damage through various mechanisms. Eventually, If neglected, the disease can advance, causing chronic damage to organs such as the kidneys, heart, lungs, and central nervous system (CNS), and may even prove to be fatal [7-10].

Gender disparities related to disease susceptibility are not uncommon. Several types of cancers and autoimmune disorders are more commonly seen in females than males. Besides, the clinical course and outcomes of diseases also reveal gender disparities as evidenced during the coronavirus disease 2019 (COVID-19) pandemic - caused by the severe acute respiratory syndrome coronavirus-2 (SARS-CoV-2) - wherein infected males developed more serious adverse effects compared to females [11]. This has prompted research to understand gender variations and devise gender-based treatment and management strategies [12,13]. Similarly, women are 10 times (9:1) more susceptible to SLE than men. Furthermore, there is evidence of variable clinical presentations, pathogenesis, and disease outcomes between females and males [14,15]. Although this predisposition is attributed to several factors including genetic susceptibility and environmental factors, a more comprehensive understanding of the mechanisms underlying the phenomenon is essential [16].

In developing nations, like India, the prevalence and type of infection associated with SLE varies significantly, with endemic diseases such as tuberculosis posing a greater risk [17]. In India, there is insufficient data on the overall risk of infection in SLE patients. Patients with SLE are at a significant risk of infection, which remains a leading cause of death in this population. This infection risk can be due to the pathophysiology of SLE; corticosteroids and immunosuppressive medication also increase the risk of infection [18].

Infections in SLE patients are usually bacterial in origin, such as respiratory infections caused by *Streptococcus pneumoniae*, urinary tract infections (UTIs) associated with *Escherichia coli*, and skin infections due to *Staphylococcus aureus *[19]. SLE patients are more likely to contract microbial diseases such as hepatitis B and C, tuberculosis, cryptococcosis, and Pneumocystis jiroveci pneumonia (PJP) since their cell-mediated immunity is depleted [20-22]. Herpes zoster is common among people aged over 50 years and those on immunosuppressants such as cyclophosphamide, azathioprine (AZA), or high-dose prednisone [23]. Cytomegalovirus (CMV) infects more than 90% of SLE patients. Tuberculosis is the most common infection in SLE patients. Lower respiratory tract infections (LRTIs) constitute 26.6% of infections and SLE patients have a 27.65 times higher risk of PJP than the general population [24].

Distinguishing SLE flares from infections is difficult because of overlapping symptoms such as fever and fatigue, which necessitates a thorough differential diagnosis. Despite advancements in the diagnosis and treatment of SLE, there is an increasing concern about the morbidity and mortality associated with the disease [25]. Therefore, enhancing our understanding of the pathogenesis, clinical course, and complications of SLE and the underlying mechanisms could facilitate the development of better management and therapeutic strategies.

## Review

SLE is a complex disease associated with genetic inclination potentially revealed through inheritance patterns. Hundreds of gene polymorphisms and mutations are linked to the risk of developing SLE. Individuals with Klinefelter's syndrome were found to be at increased risk of developing SLE. Further, it was identified that the gene responsible for SLE is coded on the X chromosome while the exact gene has not been identified yet. This is probably why females are 10 times more predisposed to SLE than males [4]. 

**Figure 1:**
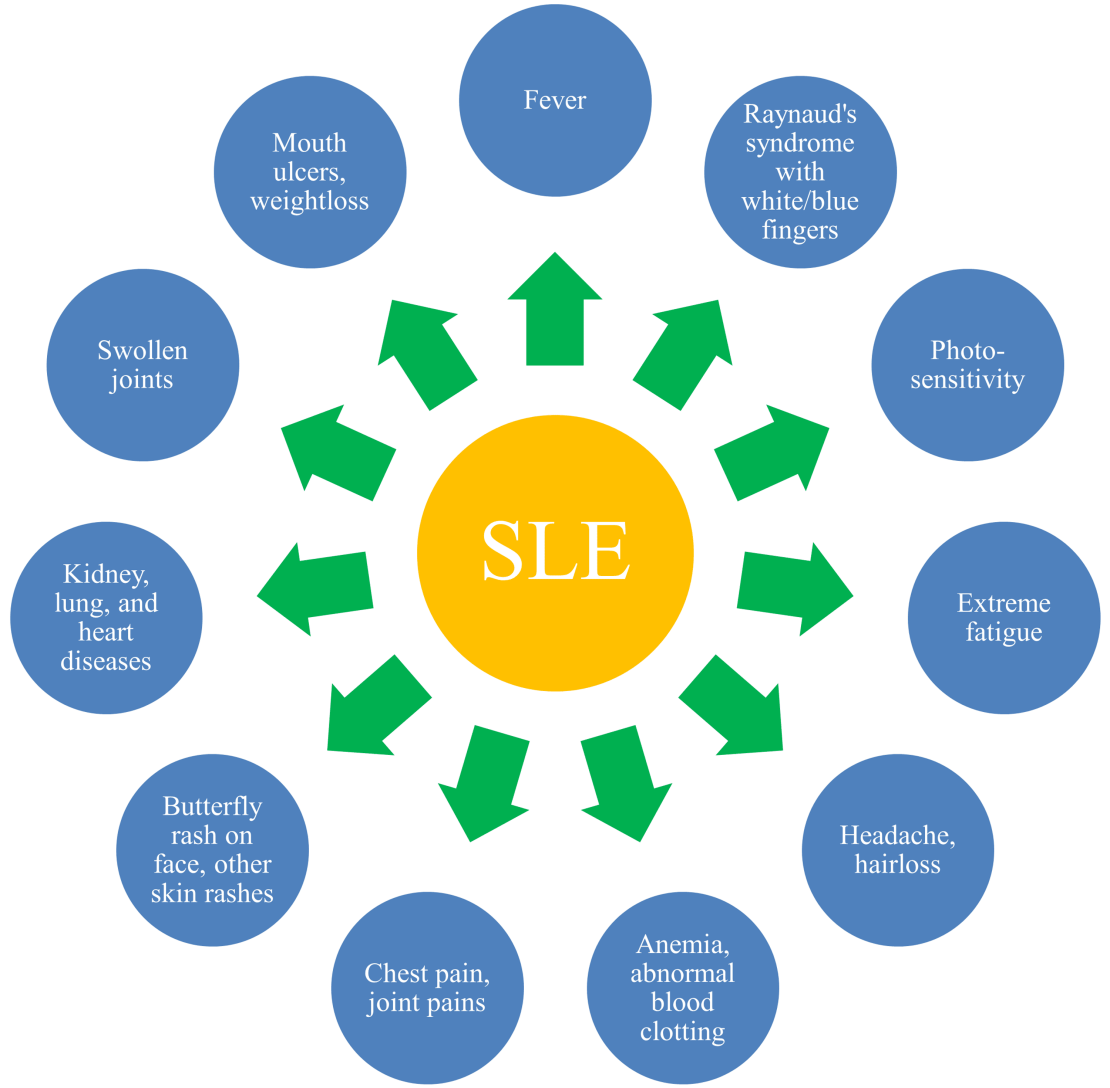
Clinical manifestations of SLE Image credit: Venkataramana Kandi SLE: systemic lupus erythematosus

Several factors could explain the gender-based variations in the prevalence of SLE manifestations. Hormonal influences, genetic differences, and immune responses may play a role. Epidemiological studies show that the prevalence of SLE varies by country and ethnic group, indicating that besides hormonal and genetic predisposition, regional and environmental factors play a role in the development of SLE [26]. While SLE is more common in women than in men, male patients are likely to develop more severe diseases [27]. There is a significant female predominance in disease incidence (female-to-male ratio: 9:1) [28]. However, research indicates that males have a poor prognosis with SLE. Understanding the gender-based clinical manifestations of SLE is important for establishing separate treatment strategies and improving patient outcomes. SLE patients present with multiple symptoms (Figure 1).

Common manifestations in female SLE patients

Female patients with SLE most commonly develop clinical manifestations like alopecia (60.90%), photosensitivity (60.70%), oral ulcers (51.00%), arthritis (74.20%), malar rash (55.60%),​ discoid lupus (20.40%), and low complement component 3 (C3) activities (51.10%) [29]. Low complement levels, specifically C3, are found in 51.10% of patients, indicating increased disease activity and immune complex formation [15]. This shows the diverse and severe clinical manifestations faced by female SLE patients compared to male patients (Table 1).

**Table 1:** Common clinical manifestation in female SLE patients compared to their male counterparts This table has been created by the authors C3: complement component 3; SLE: systemic lupus erythematosus

Clinical manifestation/evidence	Females	Males
Alopecia	60.90%	30-40%
Photosensitivity	60.70%	25-35%
Oral ulcers	51.00%	20-30%
Arthritis	74.20%	40-50%
Malar rash	55.60%	20-30%
Discoid lupus	20.40%	10-20%
Low C3 activities	51.10%	30-40%

Common manifestations in male SLE patients

Although the prevalence of SLE is lower in males than in females, males demonstrate unique manifestations like renal involvement (54.20%), serositis (56.00%), pleuritis (47.10%), thrombocytopenia (20.20%), elevated anti-double-stranded deoxyribonucleic acid (dsDNA) antibody activities (66.10%), and pericarditis (27.30%) (Table 2) [30,31].

**Table 2:** Common manifestations in male SLE patients compared to their female counterparts This table has been created by the authors dsDNA: double-stranded deoxyribonucleic acid; SLE: systemic lupus erythematosus

Clinical manifestation/evidence	Males	Females
Renal involvement	54.20%	30-35%
Serositis and pleurisies	56.00%	30-40%
Pleuritis	47.10%	20-30%
Thrombocytopenia	20.20%	15-20%
Anti-dsDNA antibodies	66.10%	45-55%
Pericarditis	27.30%	10-20%

Mechanisms underlying gender bias in SLE

Autoimmune disorders are more prevalent in females than in males, possibly due to sex hormones such as estrogen [32-34]. Estrogen is the main hormone that plays a role in the mechanism of autoimmune disorders, even though estrogen levels do not significantly vary between affected and unaffected females [16]. Estrogen acts by binding to estrogen receptors (ERα and ERβ).

ERα plays a key role in autoimmune disorders like SLE, influencing innate and adaptive immune responses. It boosts the helper thymus cell type 2 (Th2) immune response (dominant humoral responses) and increases cytokine production, such as interleukins (ILs) 4, 10, and others [35]. This receptor is widely expressed in immune cells such as thymocytes, thymus (T) and bone marrow (B) cells, dendritic cells, and macrophages. Estradiol, a kind of estrogen, can stimulate cytokine production and effector cell activation, resulting in the proliferation of M2-type macrophages and myeloid-derived suppressor cells. Estrogen and prolactin can also stimulate autoreactive B cells, leading to immunological tolerance failure and increased autoantibody production, contributing to greater rates of autoimmune diseases in females [35].

**Figure 2:**
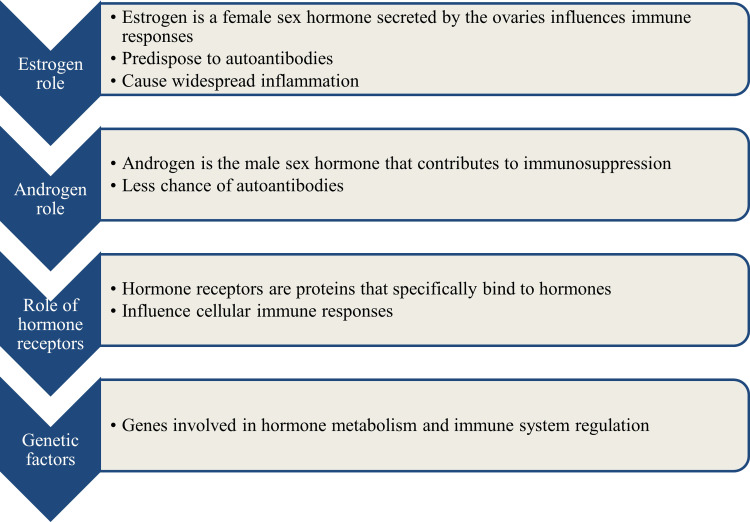
Factors influencing gender-based differences in SLE Image credit: Venkataramana Kandi SLE: systemic lupus erythematosus

Thus, the association between sex hormones that increase serum levels of some cytokines and the estrogen receptor may be important in disease development [36]. Further research studies to find the exact mechanisms behind these gender-based differences are currently ongoing (Figure 2).

Genetic susceptibility to SLE

Familial association and high occurrence chances in identical twins support the genetic link in SLE, despite the lack of a clear inheritance pattern. The genetic background of SLE involves polymorphisms in various genes, especially those related to immune regulation, apoptosis, and inflammation. Recent studies have shown that identical twins may have a concordance rate of up to 50% [37,38]. Approximately 100 genetic loci have been linked to polygenic SLE, with the majority containing common polymorphisms. More than 30 genes are known to generate monogenic versions of the illness or related symptoms [39]. These genetic variables are primarily linked to the activation of the immune response to foreign and self-antigens and the stimulation of the innate and adaptive immune systems [40].

Particular rare gene variants increase the chance of developing SLE, including deficiencies in early complement components like C1q and C1r/C1s (>90% risk), C4 (about 50% risk), and C2 (roughly 20% risk) [41]. The significant genetic predisposition is located at the major histocompatibility complex (MHC) locus, which contains genes responsible for antigen presentation, such as class I human leukocyte antigen (HLA) types (HLA-A, B, and C) and class II HLA molecules (HLA-DR, DQ, and -DP). HLA gene variants such as HLA-DRB1, HLA-DR2, HLA-DR3, tumor necrosis factor alpha-induced protein 3 (TNFAIP3), signal transducer and activator of transcription 4 (STAT4), and toll-like receptor 7 (TLR7) have also been associated with a predisposition to SLE [42]. Three prime repair exonuclease 1 (TREX1) gene mutations have been reported at a higher frequency (0.5-3%) in SLE patients [42].

A rare, inherited form of SLE has an autosomal recessive inheritance pattern, which suggests that both copies of the gene in each cell have pathogenic variations. The parents of an individual with an autosomal recessive disorder each have one copy of the mutated gene, although they usually do not exhibit symptoms of the condition [36,37]. Sustained genetic analysis of SLE patients will further consolidate known genetic associations and identify causal alleles across multiple ethnicities. Next-generation sequencing studies may identify rare variants with moderate risk of SLE, which could provide insights into disease-relevant cell types and signaling pathways. The discovery of more SLE-risk variants could provide more diagnostic and prognostic biomarkers for the disease.

Immune responses in SLE

**Figure 3:**
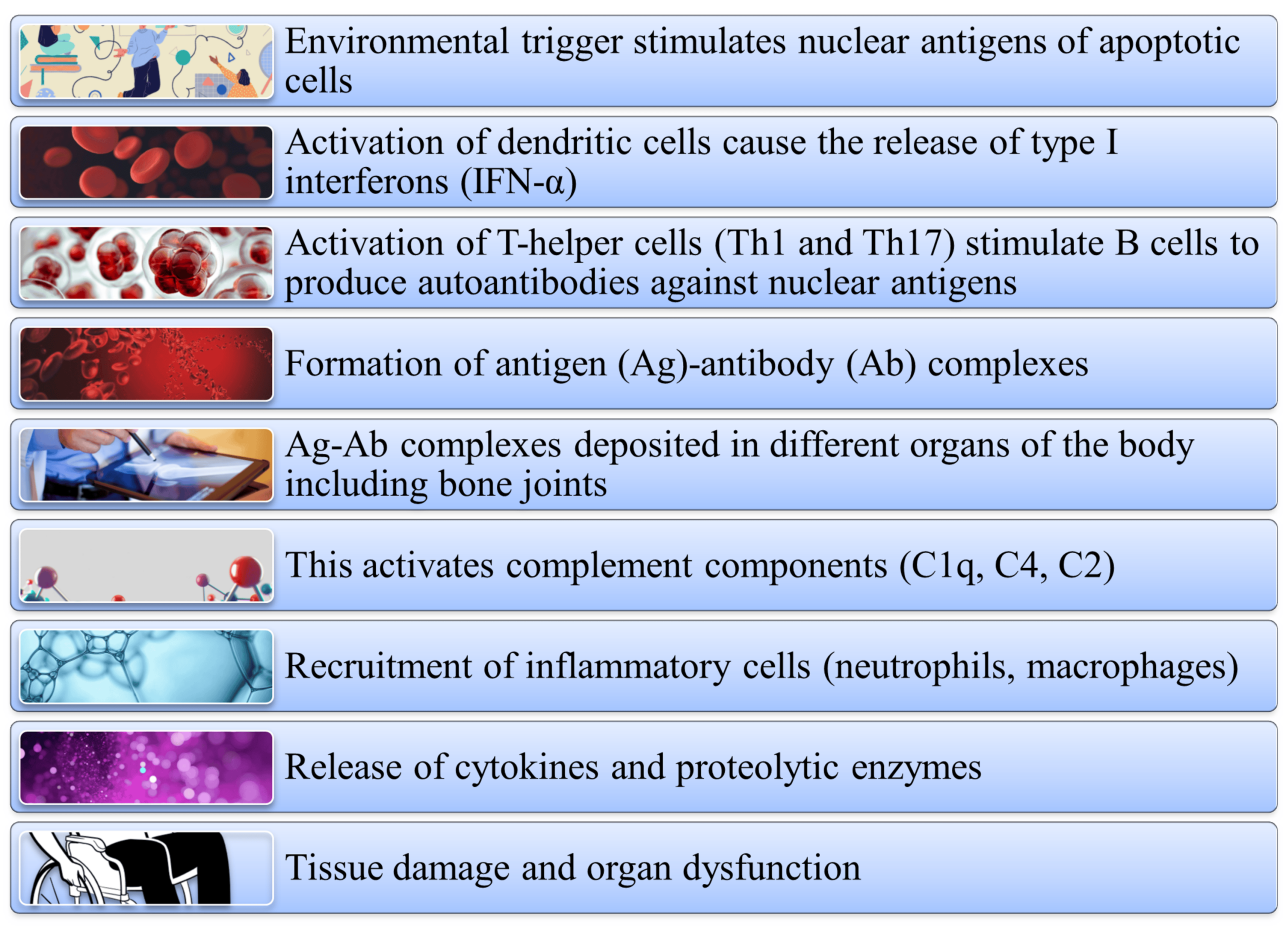
Pathogenesis of SLE Image credit: Venkataramana Kandi SLE: systemic lupus erythematosus

The pathogenesis of SLE is influenced by innate immunity and adaptive immune responses. SLE predisposes people to develop autoantibodies that trigger the activation of innate immunological responses releasing inflammatory cells like neutrophils, macrophages, and other cells. This results in the production of type I interferons (IFN-alpha). Furthermore, the immune cells of the adaptive immune responses like the cluster of differentiation 4 (CD4+) T and B cells are stimulated to initiate Th1 (dominant cellular responses) and Th17 (both cellular and humoral responses) responses against the autoantibodies. This process causes the formation of antigen (Ag) and antibody (Ab) complexes in SLE patients. These complexes spread to different organs through blood circulation and are deposited in sites like bone joints. The Ag-Ab complexes activate the complement system and its components like C1q, C2, and C4. The complement activation releases cytokines and other inflammatory substances like proteolytic enzymes, leading to the lysis of cells, tissue damage, and organ dysfunction (Figure 3).

Susceptibility to SLE-associated pathogenesis was evident among patients with hereditary deficits in early complement components including C1q, C2, and C4. Complement deficiency makes it more difficult for the mononuclear phagocytes to remove circulating immune complexes, which leads to the deposition of these complexes in various tissues and organs [43]. Deficiency of C4 or specific complement receptors increases the risk of developing autoimmune disorders similar to SLE. Numerous mechanisms have been proposed, such as loss of B-cell self-tolerance and inability to remove immunological complexes. Additionally, it has been shown that a lack of C1q leads to impaired phagocytic clearance of apoptotic cells. Apoptosis is a normal process for many malfunctioning and altered cells to undergo self-death. Failure to trigger apoptosis may activate immune responses against the nuclear components as observed in SLE [43].

In a recent study, dysregulated immunological responses in SLE were attributed to the development of abnormal B cells in the bone marrow. It was noticed that people suffering from SLE demonstrated two types of B cells, the normal early B cells (EB^nor^) and abnormal/defective early B cells (EB^lo^). People who develop defective B cells suffer from a severe form of the disease compared to those who generate normal B cells [44].

SLE patients develop a condition called macrophage activation syndrome (MAS), a form of cytokine storm in which they suffer from disease complications that are difficult to differentiate from SLE flares and infection-associated complications. MAS predisposes SLE patients to poor clinical outcomes [45]. Besides, it has been observed that the T lymphocytes suffer from metabolic dysregulation creating an atmosphere of autoreactivity and inflammation [46,47]. COVID-19 vaccination among SLE patients revealed impaired innate and adaptive immune responses confirming the influence of SLE on the immune system [48].

Gender influence in the pathogenesis of arthritis

**Figure 4:**
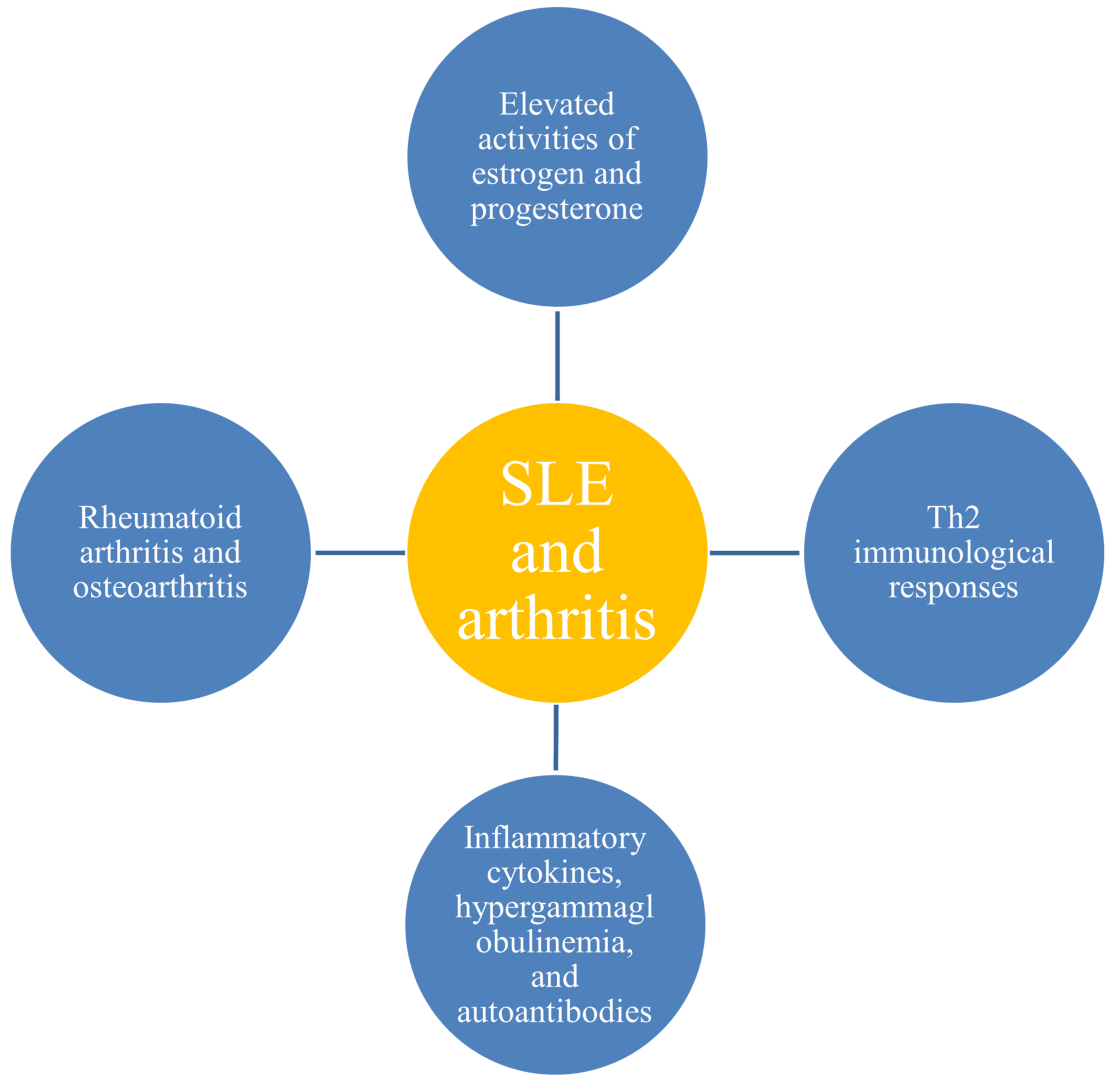
Pathogenesis of arthritis in females suffering from SLE Image credit: Venkataramana Kandi SLE: systemic lupus erythematosus

Arthritis is more prevalent in women (74.2%) than men with SLE. This higher incidence in women is likely due to increased expression of inflammatory cytokines, which may be influenced by hormonal variables such as estrogen [49]. Increased estrogen and progesterone can trigger a Th2-dominated immunological response. This leads to excessive cytokine stimulation comprising ILs 4, 5, and 13, affecting bones and joints. Further, Th2 responses enhance immunoglobulin synthesis leading to hypergamaglobulinemia. Women develop pain sensitivity and chronic conditions like rheumatoid arthritis and osteoarthritis. The affected persons suffer from greater functional disabilities and disease severity (Figure 4).

In contrast, men with SLE experience mild arthritis, probably due to lower estrogen and progesterone activities and normal testosterone activities. Additionally, in males, the Th1 immunological responses dominate, and this results in the release of gamma IFN (IFN-γ), TNF, and IL-2.

SLE and renal involvement

**Figure 5:**
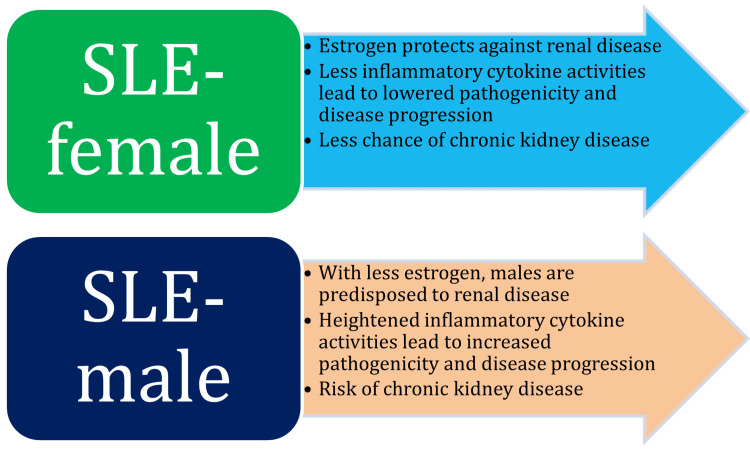
Comparison of renal involvement in males and females with SLE Image credit: Venkataramana Kandi SLE: systemic lupus erythematosus

Renal involvement, especially lupus nephritis, is more prevalent and severe in males than in women with SLE [49]. The risk factors for chronic kidney disease (CKD) among lupus nephritis patients include type 2 diabetes mellitus (T2DM), hypertension, obesity, smoking, sodium and protein-rich diet, pregnancy, aging, genetics, and others [50]. Accumulation of programmed cell death ligand 1 (PD-L1)-sensitized basophils, the release of IL-4, and autoreactive T follicular helper (TFH) cells in SLE patients trigger disease progression and lupus nephritis [51,52]. About 61% of males with SLE have renal involvement, compared to 32% in women. Men with SLE are also much more likely to advance to end-stage renal disease (ESRD) compared to women (with a hazard ratio of 5.1) [15]. Estrogen plays a protective role in the health of the kidneys. Males with lowered activities of estrogen suffer from increased cytokine activities leading to inflammation, nephritis, and CKD compared to females (Figure 5).

SLE and infections

Considering the influence of SLE on immunological responses and because the treatment of SLE involves the use of immunosuppressive drugs, there is a potential risk of infections in SLE patients [53]. More recently, COVID-19 has seen a high predisposition of SLE patients to COVID-19 and increased susceptibility of COVID-19 patients to develop SLE [54]. This explains the complex relationship of SLE with infection and vice-versa. A cross-talk between COVID-19 and SLE was suggested through IFNs and IFN I/II receptors, creating a pro-inflammatory atmosphere by accumulating (IL)-6/10, TNF-α, and IFN-γ among others [55]. Factors like SLE disease severity, immune dysregulation, immunosuppressive drugs, and organ damage could be attributed to infectious disease susceptibility among SLE patients [56]. Besides, infections caused by some microbes like human CMV and Epstein-Barr virus (EBV) could fasten the progression of autoimmune diseases like SLE [57,58]. Reactivation of EBV was found to trigger SLE flares, especially affecting women [59].

Experiments in mice have revealed that EBV infection activates STAT3, inhibits cellular musculoaponeurotic transcription factor (c-Maf) expression, and releases cytokines like IFN-α, and Th17-type immune cells. This affects the CD4+T regulatory (Treg) cells and results in the dysregulation of immune responses causing SLE [60]. Gender-bias-related clinical outcomes in COVID-19 patients revealed severe infections and adverse outcomes in men compared to women. This was implicated due to the variable immune responses associated with hormonal effects [61]. Cytokine storm syndrome following infection with bacteria like Brucella, *Leptospira*, *Legionella*, *Mycoplasma pneumoniae*, and *Mycobacterium tuberculosis *among others was found to be associated with the risk of disease progression in SLE patients [62]. 

**Figure 6:**
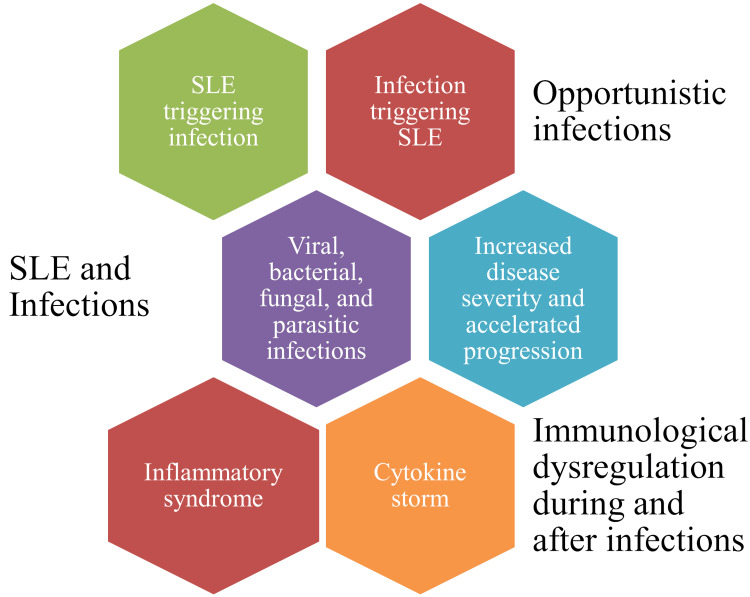
Infection and SLE disease pathogenicity Image credit: Venkataramana Kandi SLE: systemic lupus erythematosus

Post-COVID-19, diagnosis of multisystemic inflammatory syndrome (MIS-C) in an immunocompetent child suggests the potential role of SLE in modulating the immune system during and after infections [63]. Furthermore, SLE was diagnosed in a child following infections with the severe acute respiratory coronavirus-2 (SARS-CoV-2) and EBV suggesting viral infections as potential triggers of SLE [64]. Infection (lophomoniasis) by a rare parasitic protozoan like *Lophomonas blattarum* in an SLE patient confirms the role of opportunistic infections in these patients [65]. Progressive multifocal leukoencephalopathy (PML) in an SLE patient with the John Cunningham/JC virus (JCV) virus infection of the brain and lymphocytopenia confirms the role of rare viral pathogens in complicating the SLE disease course [66] (Figure 6).

Treatment and management of SLE

Clinicians find treating SLE challenging due to its diverse and unpredictable clinical profile evident in patients presenting with non-infectious and infectious complications. Besides, the pathogenesis of SLE involving the dysregulated immune system further affects patient management. There is no specific therapeutic drug to treat SLE. Hydroxychloroquine (HCQ) is effective in managing SLE patients due to its protective effect enabling a lower burden of antigens, autoantibodies, and IFNs. HCQ reduces SLE flares, lowers skin, and musculoskeletal complications, and contributes to a better quality of life (QoL). Glucocorticoids (GCs) like prednisone and methylprednisolone are recommended in appropriate doses to manage SLE patients with lupus nephritis, and blood and CNS disorders [67].

According to the guidelines recommended by the European Alliance of Associations for Rheumatology (EULAR), the first-line treatment strategies include HCQ, GC, and calcineurin inhibitors (CNI) for mild SLE. For moderate SLE, AZA, methotrexate (MTX), mycophenolate mofetil (MMF), and CNI are recommended as the first-line treatment. Cyclophosphamide (CYC) is recommended to treat severe forms of SLE. Treatment-unresponsive or refractory SLE patients are managed with MTX, AZA, belimumab (BEL), anifrolumab, rituximab (RTX), and voclosporin (VSC) [68-70].

Currently, clinical trials including Belimumab International Study in Lupus Nephritis (BLISS-LN) and Aurinia Renal Response in Active Lupus With Voclosporin (AURORA-1) are underway to find more efficient treatment and management strategies for lupus nephritis (LN) [71]. Since SLE affects different organs, biomarkers like autoantibodies against C1q (anti-C1qAb) have been suggested as appropriate alternatives to the management of SLE [72]. The application of chimeric antigen receptor (CAR) T-cell therapy to manage refractory autoimmune diseases like SLE is being explored. Despite the usefulness of CAR-T cell therapy in treating SLE, the dosage, safety, and efficacy of this treatment modality require additional studies [73]. Novel treatment strategies alternative to traditional analgesics have been under investigation to treat SLE-associated pain [74]. Although some treatments are available, medication adherence has been a challenge for SLE patients affecting their QoL [75].

SLE patients are predisposed to opportunistic infections caused by bacteria, viruses, parasites, and fungi. However, currently, there are no recommendations/strategies for the treatment and management of infectious diseases in SLE patients. Furthermore, SLE treatments may interfere with infectious disease management. The efficacy of prophylactic antibiotic therapy and immunization/vaccination has been previously debated [76]. Non-pharmacological interventions like vitamin D supplementation, dietary modifications, and other preventive measures including avoiding ultraviolet light exposure limiting GC dosage, and active management of co-morbid conditions like diabetes, hypertension, and others have been suggested to minimize the morbidity and mortality associated with SLE [67].

## Conclusions

Gaining a better understanding of gender-specific SLE symptoms helps physicians make a precise diagnosis of the condition. Clinicians should be aware that certain symptoms of SLE are more prevalent among women, whereas men may experience atypical presentations. Female patients frequently present with alopecia, photosensitivity, oral ulcers, arthritis, malar rash, high lupus anticoagulant levels, and low C3 levels. However, males are more likely to have renal involvement, serositis, pleuritis, thrombocytopenia, and high anti-dsDNA antibody levels. New evidence suggests that sex hormones, genetic predisposition, and immune system variability contribute to the gender discrepancy in SLE disease pathogenesis.

This review highlights the significant gender-based dissimilarities in the clinical manifestations of SLE and the potential immunopathogenic mechanisms behind them. SLE patients are at a higher risk of developing infectious diseases and related complications that influence disease progression and clinical outcomes. Our findings shed light on the complexity of SLE and the importance of knowing gender-specific characteristics to help clinicians make better decisions and personalize treatment approaches. Personalizing management measures by gender could help focus on specific symptoms and risk factors, resulting in better long-term results and improved QoL.
